# Supplementary material for: Significant Southern Hemisphere contribution to the Indonesian Throughflow over the last 800,000 years
Source: Nat Commun. 2026 Apr 14;17:3484. doi: 10.1038/s41467-026-71786-1 (PMC13079854; doi:10.1038/s41467-026-71786-1)
Supplement: Supplementary file 1 — Supplementary Information [file 41467_2026_71786_MOESM1_ESM.pdf]

Supplementary Information

Significant Southern Hemisphere contribution to the Indonesian Throughflow over the last 800,000 years

Markus Kienast[1†], Martina Hollstein[2*], Nadine Lehmann[1,3], Patrick A. Rafter[4], Ziye Li[5], Min-Te Chen[6], Mahyar Mohtadi[3,7*]

1: Department of Oceanography, Dalhousie University, Halifax, NS, Canada

† Deceased 12 July 2025

2: MARUM - Center for Marine Environmental Sciences, University of Bremen, Bremen, Germany

3: Institute for Marine and Antarctic Studies, University of Tasmania, Hobart, Australia

4: College of Marine Science, University of South Florida, St. Petersburg, FL, USA

5: College of Marine Geosciences, Ocean University of China, Qingdao, China

6: Institute of Earth Sciences, National Taiwan Ocean University, Keelung, Taiwan

7: Faculty of Geosciences, University of Bremen, Bremen, Germany

* Corresponding authors, mhollstein@marum.de, mmohtadi@marum.de

This file includes:

Supplementary Notes

Supplementary Table 1

Supplementary Figures 1-6

Supplementary Notes

Bulk sedimentary $\delta^{15}N$

Despite overwhelming evidence that sedimentary $\delta^{15}N$ records provide a reliable monitor of changes in the marine N cycle, especially in continental margin settings (see syntheses by refs. 1,2), bulk sedimentary $\delta^{15}N$ has come under scrutiny recently because of evidence of diagenetic overprint[3]. In line with prior studies of WEP $\delta^{15}N$[4-10], our interpretation explicitly hinges on the assumption that bulk sedimentary $\delta^{15}N$ faithfully records past variations in $\delta^{15}N_{(nitrate)}$. We justify this assumption by four independent lines of evidence. (a) There is an irrefutable consistency overall between water column and sedimentary $\delta^{15}N$ composition in the WEP; (b) the distinct hemispheric end-member composition observed today is reproducible during the last 25 kyrs in multiple sedimentary $\delta^{15}N$ records north and south of the equator (Fig. 1b), despite vastly different sedimentation rates, water depths, and overall hydrographic and sedimentological settings of the individual core sites (Supplementary Table 1); (c) a recent study[11] demonstrates that past variations in $\delta^{15}N$ along the equatorial Pacific are captured identically in bulk sedimentary and foraminifera-bound $\delta^{15}N$, and, lastly, (d), the strong obliquity signal in the Banda Sea $\delta^{15}N$ (Figs. 2, 3) implies a far-field control (Fig. 3) that is inconsistent with a more local forcing of WEP $\delta^{15}N$.

In addition, terrigenous nitrogen contamination potentially influences the $\delta^{15}N$ signal. To verify the marine origin of bulk organic matter, we measured C/N ratios and the carbon isotopic composition of bulk organic matter ($\delta^{13}C_{org}$) on multiple samples throughout the MD01-2380, because C/N ratios and $\delta^{13}C_{org}$ from marine and terrestrial sources are remarkedly different. Typical C/N ratios of marine sources range between 4 and 10, typical $\delta^{13}C_{org}$ values are between -22 and – 20 ‰.[12]. The average C/N ratio (8.6) and $\delta^{13}C_{org}$ (-20.9 ‰) of the MD01-2380 samples clearly indicate a marine origin of the bulk organic matter. The regional consistency in $\delta^{15}N$ values across the WEP cores, despite their distinct environments and sedimentation rates, additionally reinforces the conclusion that $\delta^{15}N$ is independent of terrigenous input.

Indonesian Throughflow

While the majority of the ITF circulates through the Banda Sea, a small portion flows through the Makassar Strait and exits the Indonesian Seas through the Lombok Strait without entering the Banda Sea. The most accurate data measured from different outflow passages of the ITF

during the INSTANT campaign (2004-2006) indicate that this portion makes up 17% of the total volume[13]. Another portion flows through the Makassar and eastern passages before exiting through the Ombai Strait. This translates to 33% of the total ITF. It is uncertain how much of this portion transits through the central Banda Sea[14,15]. The remainder of the ITF enters through the eastern passages, flows through the Banda Sea before exiting into the Indian Ocean via the Timor Passage. Altogether, estimated 50 to 83% of the ITF circulate through the Banda Sea before exiting through the Ombai Strait and Timor Passage. Assuming a total volume transport of the ITF of 15 Sv[16], this would translate to 7.5 – 12.5 Sv.

Supplementary Table 1. Summary of the $\delta^{15}N$ records included in the Northern and Southern Hemisphere WEP stacks. Calculated sedimentation rates refer to the last 25 kyrs.

**Site**	**Longitude [°N]**	**Latitude [°E]**	**Water depth [m]**	**Stratigraphy**	**$\delta^{15}N$ record**	**Average sedimentation rate [cm/ka]**
MD06-3067	6.51	126.50	1574	Bolliet et al., 2011[17]	Kienast et al., 2008[7]; this study	18
MD98-2181	6.3	125.83	2114	Stott et al., 2021[18]	Kienast et al., 2008[7]; this study	91
MD98-2177	1.4	119.08	968	Khider et al., 2011[19]; Stott, unpublished	This study	39
MD01-2386	1.13	129.79	2816	Jian et al., 2020[20]	Jia and Li, 2011[10]	45
MD97-2138	-1.25	146.23	1960	de Garidel-Thoron et al., 2007[21]	This study	11
U1486	-2.37	144.60	1332	Lambert et al., 2022[9]	Lambert et al., 2022[9]	6
MD05-2920	-2.86	144.53	1849	Tachikawa et al., 2014[22]	This study	23
MD98-2162	-4.69	117.9	1855	Jian et al., 2022[23]	This study	61

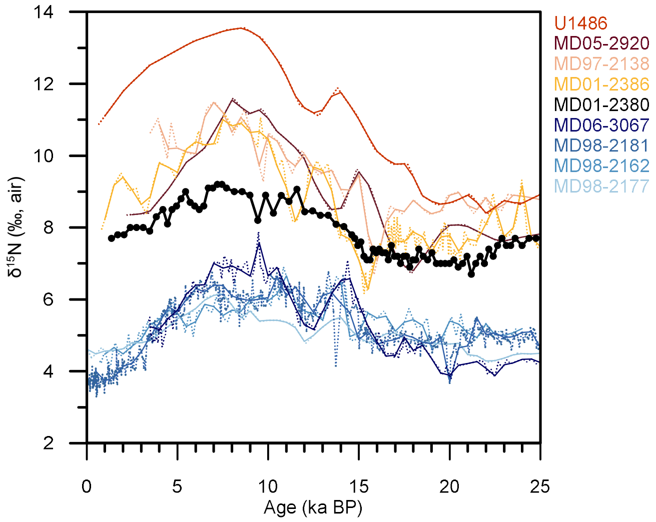

Supplementary Fig. 1. Synthesis of high-resolution sedimentary $\delta^{15}N$ records from the WEP covering the last 25,000 yrs. All $\delta^{15}N$ records are shown on their independent age models (see Supplementary Table 1). U1486[9] (red), MD05-2920 (dark red), MD97-2138 (pink), MD01-2386[10] (yellow), MD06-3067 (dark blue), MD98-2181 (ice blue), MD98-2162 (light blue), and MD98-2177 (lightest blue). See Fig. 1 for time-dependent mean and standard deviation.

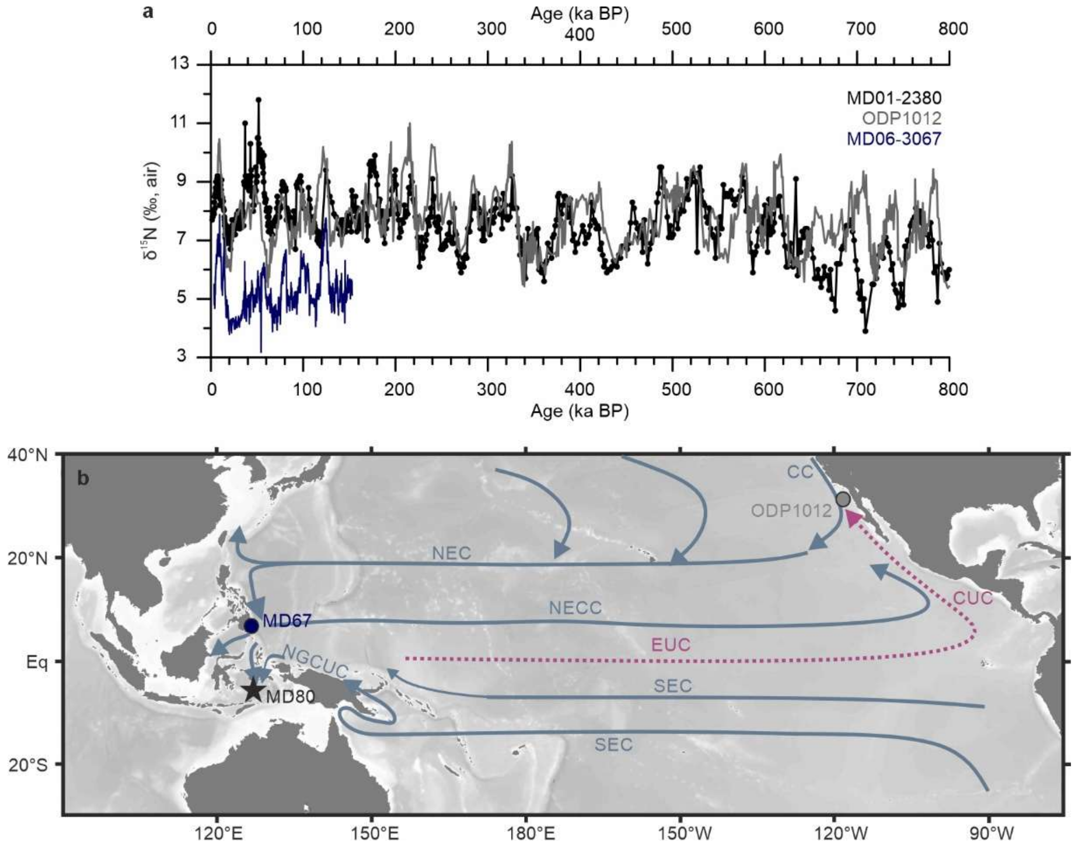

Supplementary Fig. 2. Comparison of bulk sedimentary $\delta^{15}N$ records and relation to the regional ocean circulation. **(a)** Record of MD01-2380 from the Banda Sea (black, this study) compared to the records from site MD06-3067 off Mindanao (blue, ref. 7, this study) and from the California Margin site ODP1012 (grey, ref. 24), on their original age models and on the same $\delta^{15}N$ axis. Note the remarkable agreement in $\delta^{15}N$ between the California Margin and the Banda Sea. The $\delta^{15}N$ off Mindanao, at the entrance of the ITF shows the identical temporal variability but is offset by ~2.5‰ from the former two records. **(b)** Bathymetric map of core sites. Schematic of major surface and subsurface currents adopted from ref. 25: CC – California Current, CUC – California Undercurrent, EUC – Equatorial Undercurrent, NEC - North Equatorial Current, NECC – North Equatorial Countercurrent, NGCUC – New Guinea Coastal Undercurrent, SEC – South Equatorial Current. The map was generated using ODV[26].

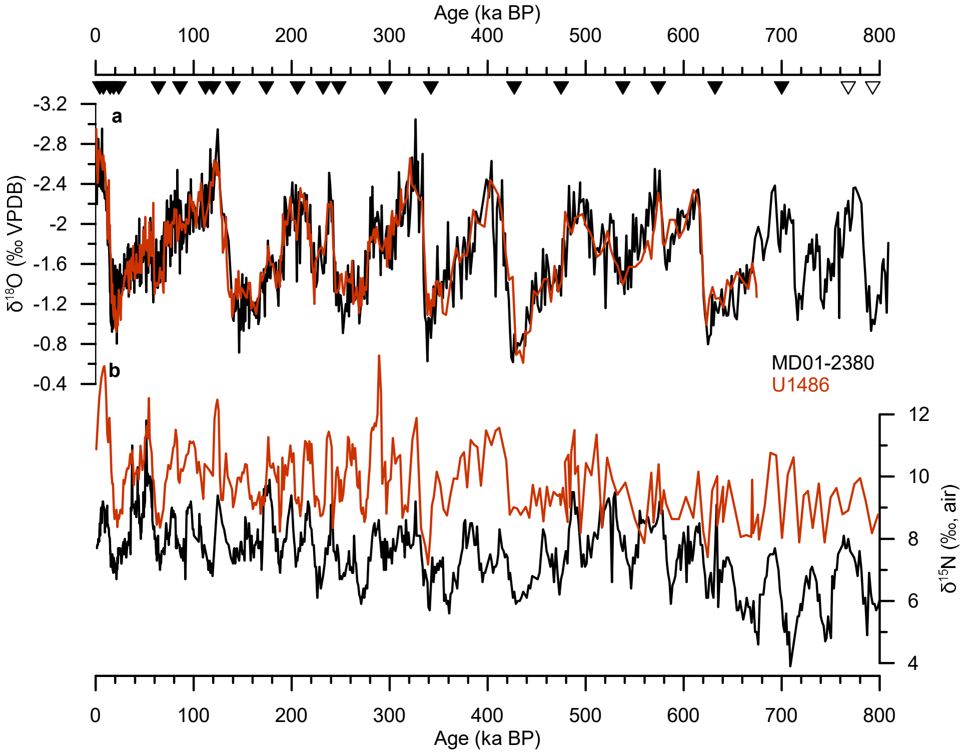

Supplementary Fig. 3. **Comparison of oxygen and nitrogen isotope records.** (**a**) $\delta^{18}O$ and (**b**) $\delta^{15}N$ records of MD01-2380 from the Banda Sea (this study) compared to the records from U1486 off Papua New Guinea (data from ref. 9[9]). Tie points were defined predominantly at the boundary of marine isotope (sub-)stages, with subordinate consideration of $\delta^{15}N$ maxima and minima in MD01-2380 and U1486. As the oxygen isotope record of U1486 covers the past 750 kyrs only, the two oldest tie points are defined by aligning the $\delta^{15}N$ records.

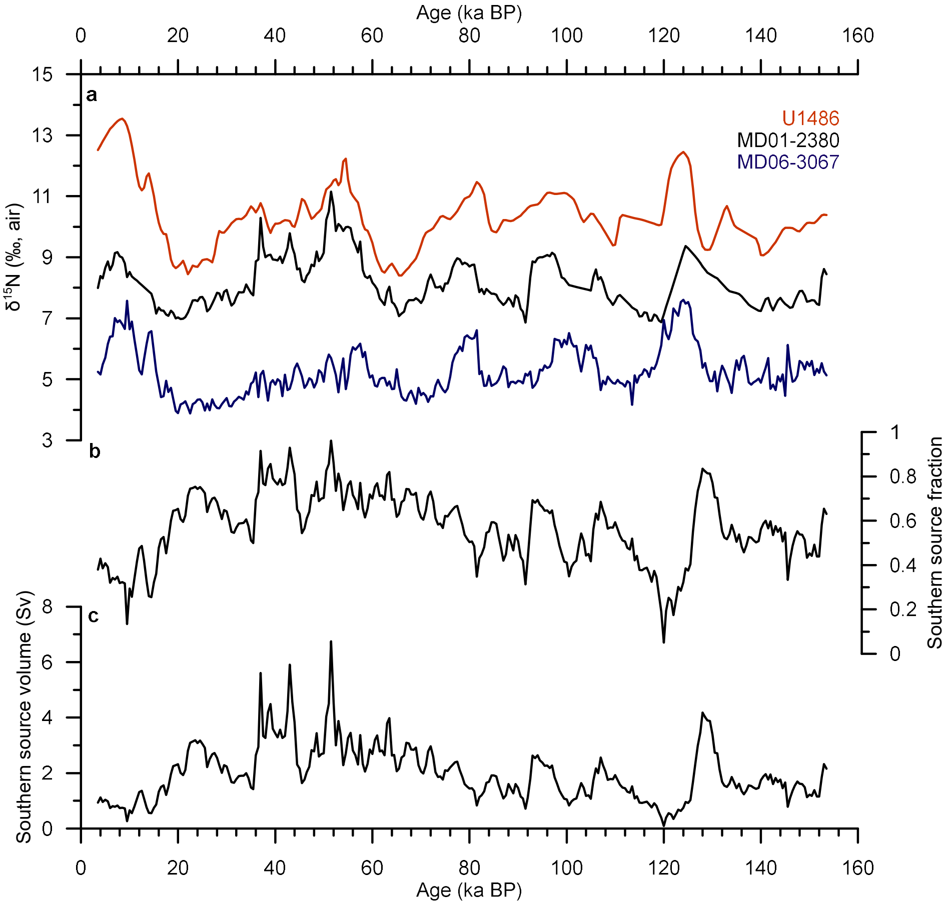

Supplementary Fig. 4. Contribution of Southern Hemisphere source waters. (a) The bulk sedimentary $\delta^{15}N$ records of MD01-2380 (black), U1486[9] (red) and MD06-3067 (blue), interpolated at time steps of 500 yrs, **(b)** the estimated fraction of Southern Hemisphere source water contribution to the Banda Sea, and **(c)** the estimated contribution of Southern Hemisphere sourced near-surface waters (0 – 300 m) to the ITF volume transport circulating through the Banda Sea.

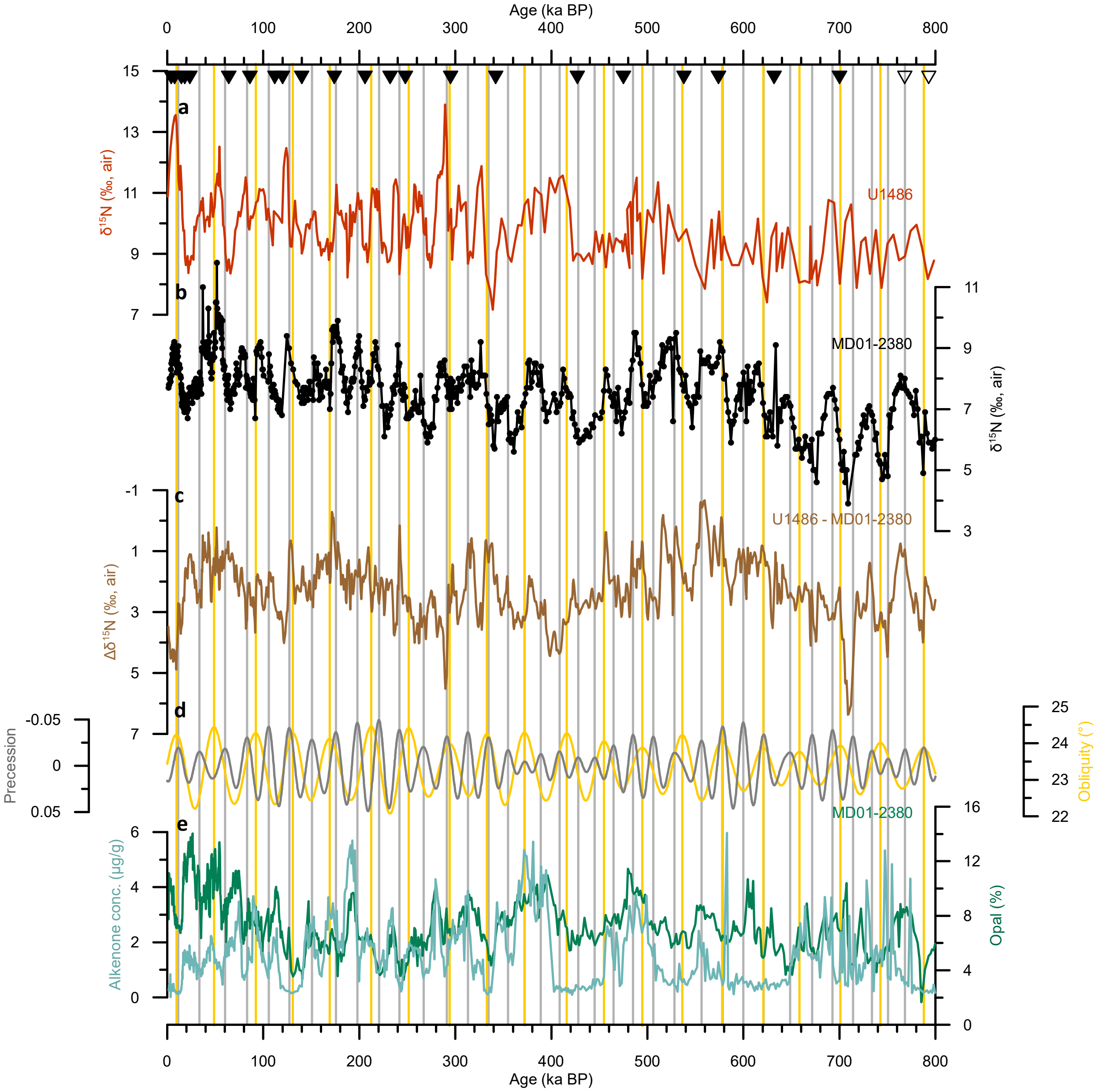

Supplementary Fig. 5. Proxy records with precession and obliquity variability. (a) $\delta^{15}N$ record of site U1486[9] (red) and **(b)** $\delta^{15}N$ record of site MD01-2380 (black), and **(c)** calculated offset (brown; see methods) between the two $\delta^{15}N$ records, $\Delta\delta^{15}N$. **(d)** Changes in obliquity (yellow) and precession (gray, inverted axis). **(e)** Percentages of biogenic opal (green) and alkenone concentration (light green) of core MD01-2380. Triangles indicate dating points of MD01-2380. Grey and yellow vertical bars highlight precession minima (y-axis is inverted) and obliquity maxima, respectively.

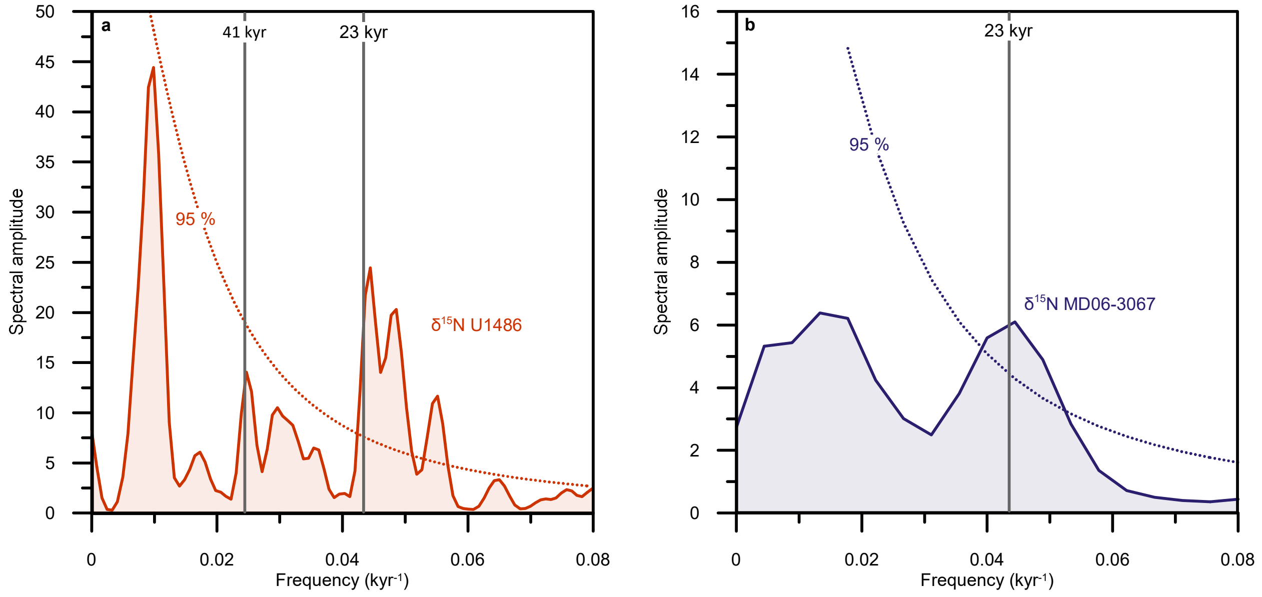

Supplementary Fig. 6. Power spectra of proxy records. Spectra of the $\delta^{15}N$ records of **(a)** U1486 and (**b**) MD06-3067 calculated with the REDFIT application[27]. Dotted lines denote the 95% confidence intervals. Vertical grey lines indicate the frequencies that correspond to precession and obliquity periodicities of 23 and 41 kyr.

References

1 Robinson, R. S. *et al.* A review of nitrogen isotopic alteration in marine sediments. *Paleoceanography* **27** (2012). https://doi.org/10.1029/2012pa002321

2 Tesdal, J. E., Galbraith, E. D. & Kienast, M. Nitrogen isotopes in bulk marine sediment: linking seafloor observations with subseafloor records. *Biogeosciences* **10**, 101–118 (2013). https://doi.org/10.5194/bg-10-101-2013

3 Altabet, M. & Francois, R. Sedimentary nitrogen isotopic ratio as a recorder for surface ocean nitrate utilization. *Global Biogeochemical Cycles* **8**, 103–116 (1994). https://doi.org/10.1029/93GB03396

4 Nakatsuka, T. *et al.* Glacial-interglacial migration of an upwelling field in the western equatorial Pacific recorded by sediment 15 N/14N. *Geophysical Research Letters* **22**, 2525–2528 (1995). https://doi.org/10.1029/95GL02544

5 Yoshikawa, C., Nakatsuka, T. & Kawahata, H. Transition of low-salinity water in the Western Pacific Warm Pool recorded in the nitrogen isotopic ratios of settling particles. *Geophysical Research Letters* **32** (2005). https://doi.org/10.1029/2005gl023103

6 Zhu, X. *et al.* Sedimentary records of nitrogen isotope in the western tropical Pacific linked to the eastern tropical Pacific denitrification during the last deglacial time. *Geo-Marine Letters* **40**, 89–99 (2020). https://doi.org/10.1007/s00367-020-00637-9

7 Kienast, M. *et al.* A mid-Holocene transition in the nitrogen dynamics of the western equatorial Pacific: Evidence of a deepening thermocline? *Geophysical Research Letters* **35** (2008). https://doi.org/10.1029/2008gl035464

8 Rafter, P. A. & Charles, C. D. Pleistocene equatorial Pacific dynamics inferred from the zonal asymmetry in sedimentary nitrogen isotopes. *Paleoceanography* **27** (2012). https://doi.org/10.1029/2012pa002367
9 Lambert, J. E. *et al.* Equatorial Pacific bulk sediment δ15N supports a secular increase in Southern Ocean nitrate utilization after the mid-Pleistocene Transition. *Quaternary Science Reviews* **278** (2022). https://doi.org/10.1016/j.quascirev.2021.107348
10 Jia, G. & Li, Z. Easterly denitrification signal and nitrogen fixation feedback documented in the western Pacific sediments. *Geophysical Research Letters* **38** (2011). https://doi.org/10.1029/2011gl050021
11 Rafter, P. A. *et al.* Persistent eastern equatorial Pacific Ocean upwelling since the warm Pliocene. *Science* (in press).
12 Meyers, P. A. Preservation of elemental and isotopic source identification of sedimentary organic matter. *Chemical Geology* **114**, 289–302 (1994). https://doi.org/https://doi.org/10.1016/0009-2541(94)90059-0
13 Sprintall, J., Wijffels, S. E., Molcard, R. & Jaya, I. Direct estimates of the Indonesian Throughflow entering the Indian Ocean: 2004–2006. *Journal of Geophysical Research: Oceans* **114** (2009). https://doi.org/10.1029/2008jc005257
14 Liang, L., Xue, H. & Shu, Y. The Indonesian Throughflow and the Circulation in the Banda Sea: A Modeling Study. *Journal of Geophysical Research: Oceans* **124**, 3089–3106 (2019). https://doi.org/10.1029/2018jc014926
15 Guo, Y. *et al.* Water sources of the Lombok, Ombai and Timor outflows of the Indonesian throughflow. *Frontiers in Marine Science* **10** (2023). https://doi.org/10.3389/fmars.2023.1326048
16 Sprintall, J. *et al.* Detecting Change in the Indonesian Seas. *Frontiers in Marine Science* **6** (2019). https://doi.org/10.3389/fmars.2019.00257
17 Bolliet, T. *et al.* Mindanao Dome variability over the last 160 kyr: Episodic glacial cooling of the West Pacific Warm Pool. *Paleoceanography* **26** (2011). https://doi.org/10.1029/2010pa001966
18 Stott, L. D., Shao, J., Yu, J. & Harazin, K. M. Evaluating the Glacial-Deglacial Carbon Respiration and Ventilation Change Hypothesis as a Mechanism for Changing Atmospheric CO2. *Geophysical Research Letters* **48** (2021). https://doi.org/10.1029/2020gl091296
19 Khider, D., Stott, L. D., Emile-Geay, J., Thunell, R. & Hammond, D. E. Assessing El Niño Southern Oscillation variability during the past millennium. *Paleoceanography* **26**, PA3222 (2011). https://doi.org/10.1029/2011PA002139
20 Jian, Z. *et al.* Half-precessional cycle of thermocline temperature in the western equatorial Pacific and its bihemispheric dynamics. *Proceedings of the National Academy of Sciences of the United States of America* **117**, 7044–7051 (2020). https://doi.org/10.1073/pnas.1915510117
21 de Garidel-Thoron, T. *et al.* A multiproxy assessment of the western equatorial Pacific hydrography during the last 30 kyr. *Paleoceanography* **22**, 10.1029/2006PA001269 (2007).
22 Tachikawa, K., Timmermann, A., Vidal, L., Sonzogni, C. & Timm, O. E. CO_2 radiative forcing and Intertropical Convergence Zone influences on western

Pacific warm pool climate over the past 400 ka. *Quaternary Science Reviews* **86**, 24–34 (2014). https://doi.org/10.1016/j.quascirev.2013.12.018
23 Jian, Z. *et al*. Warm pool ocean heat content regulates ocean–continent moisture transport. *Nature* **612**, 92–99 (2022). https://doi.org/10.1038/s41586-022-05302-y
24 Liu, Z., Altabet, M. A. & Herbert, T. D. Plio-Pleistocene denitrification in the eastern tropical North Pacific: Intensification at 2.1 Ma. *Geochemistry, Geophysics, Geosystems* **9** (2008). https://doi.org/10.1029/2008gc002044
25 Hu, D. *et al*. Pacific western boundary currents and their roles in climate. *Nature* **522**, 299–308 (2015). https://doi.org/10.1038/nature14504
26 Schlitzer, R. Ocean Data View, https://odv.awi.de. (2025).
27 Schulz, M. & Mudelsee, M. REDFIT: estimatingred-noise spectra directly from unevenly spaced paleoclimatic time series. *Computers & Geosciences* **28**, 421–426 (2002).
